# Ground-Based LiDAR Improves Phenotypic Repeatability of Above-Ground Biomass and Crop Growth Rate in Wheat

**DOI:** 10.34133/2020/8329798

**Published:** 2020-05-26

**Authors:** David M. Deery, Greg J. Rebetzke, Jose A. Jimenez-Berni, Anthony G. Condon, David J. Smith, Kathryn M. Bechaz, William D. Bovill

**Affiliations:** ^1^CSIRO Agriculture and Food, Canberra, ACT, Australia; ^2^CSIRO Agriculture and Food, Yanco, NSW, Australia; ^3^NSW Department of Primary Industries, Yanco, NSW, Australia

## Abstract

Highly repeatable, nondestructive, and high-throughput measures of above-ground biomass (AGB) and crop growth rate (CGR) are important for wheat improvement programs. This study evaluates the repeatability of destructive AGB and CGR measurements in comparison to two previously described methods for the estimation of AGB from LiDAR: 3D voxel index (3DVI) and 3D profile index (3DPI). Across three field experiments, contrasting in available water supply and comprising up to 98 wheat genotypes varying for canopy architecture, several concurrent measurements of LiDAR and AGB were made from jointing to anthesis. Phenotypic correlations at discrete events between AGB and the LiDAR-derived biomass indices were significant, ranging from 0.31 (*P* < 0.05) to 0.86 (*P* < 0.0001), providing confidence in the LiDAR indices as effective surrogates for AGB. The repeatability of the LiDAR biomass indices at discrete events was at least similar to and often higher than AGB, particularly under water limitation. The correlations between calculated CGR for AGB and the LiDAR indices were moderate to high and varied between experiments. However, across all experiments, the repeatabilities of the CGR derived from the LiDAR indices were appreciably greater than those for AGB, except for the 3DPI in the water-limited environment. In our experiments, the repeatability of either LiDAR index was consistently higher than that of AGB, both at discrete time points and when CGR was calculated. These findings provide promising support for the reliable use of ground-based LiDAR, as a surrogate measure of AGB and CGR, for screening germplasm in research and wheat breeding.

## 1. Introduction

The grain yield from cereals can be expressed as the product of above-ground biomass (AGB) at physiological maturity and harvest index (HI). The genetic progress in wheat grain yield potential during the last century has been generally associated with improvements in HI, but there is evidence that HI has now stabilised at *ca.* 0.5 (e.g., [1, 2, 3, 4, 5]), despite a theoretical maximum of 0.62 [6]. There is evidence that genetic progress in grain yield potential has been associated with increased preanthesis crop growth rate (CGR) and radiation use efficiency (RUE) from *ca.* 1980 [3, 7]. CGR for the preanthesis period is typically defined as the change in AGB between stem elongation and anthesis divided by the time interval [8]. Preanthesis CGR is important for the establishment of grain number and potential grain size [8]. Crop growth (i.e., the accumulated daily CGR) for the stem elongation to anthesis period is also defined as the product of canopy intercepted photosynthetically active radiation (PARi) and RUE [9]. Although PARi can be estimated remotely [10], it varies little under good growing conditions during the critical preanthesis stage (e.g., [4]); RUE cannot easily be estimated, so the direct estimation of CGR has added advantages. Future genetic progress in wheat grain yield potential is sought through increasing CGR whilst maintaining HI (e.g., [11, 12, 13]). AGB is typically measured destructively, by removing the plant material from a known area and determining the dry weight (e.g., [14, 15]). Such measurements made on large experiments are laborious and time-consuming and the repeatability can be low if the sample area is small. Therefore, nondestructive and high-throughput measures of AGB and CGR are important for wheat improvement programs.

Recent advances in proximal remote sensing present such an opportunity for nondestructive and high-throughput assessment of AGB and potentially grain yield (e.g., [16, 17, 18]). To this end, a particularly promising proximal remote sensing technology is LiDAR (Light Detection And Ranging) [19, 20]. LiDAR typically measures distance by illuminating a target with a laser and analyzing the reflected light. Thus, field measurements are not confounded by the ambient light conditions and the light normalization typically required for hyperspectral measurements is not required (e.g., [21, 22, 23, 24]). Further, as LiDAR is a laser-based sensor, it can penetrate gaps in the canopy which minimises the effect of occlusion when sequential scans from slightly different positions are combined. Recent studies have used LiDAR and similar laser-based sensors to quantify AGB, height, and leaf area index (LAI) in cereals primarily for precision agriculture applications [25–30]. These studies typically involve a single genotype whereby variation in crop canopy density is generated by nitrogen application and sowing rate treatments. In contrast, we are concerned with nondestructive measurement of AGB for the purpose of crop improvement through plant breeding.

LiDAR mounted on a portable buggy, alias “Phenomobile Lite,” was recently proposed for nondestructive assessment of height, ground cover, and AGB [16, 17, 31]. Two LiDAR algorithms were shown to be highly correlated with AGB across multiple samplings: 3D voxel index (3DVI) and 3D profile index (3DPI) [17]. In addition, in a study comprising eight bread-wheat cultivars grown across eight sites, LiDAR-derived volume estimates of the crop canopy were strongly associated with AGB and broad-sense heritability estimates from LiDAR were as good as and typically greater than those for AGB [32]. Whilst these studies are encouraging, further testing is required with a larger number of genotypes to better represent the likely scale encountered within a breeding program. This study, therefore, comprises up to 98 genotypes across three experiments in order to test whether the LiDAR biomass indices (3DVI and 3DPI) are consistently correlated with AGB (measured from conventional, destructive sampling) and to evaluate the phenotypic repeatability across multiple sampling events. In contrast to earlier work, we evaluate the repeatability of CGR calculated from LiDAR in comparison to CGR calculated from AGB. The evaluation of repeatability is an important step in determining the potential utility of a phenotyping method for plant breeding: the higher the repeatability, generally the greater the opportunity for genetic gain through indirect or direct selection [33].

## 2. Material and Methods

### 2.1. Field Experiments

We established three separate field experiments: Experiment 1 (GES15) in 2015 at CSIRO Agriculture and Food's Ginninderra Experiment Station (GES), Canberra, ACT, Australia (35.20°S, 149.09°E, elevation 577 m); Experiment 2 (Yan16), in 2016, and Experiment 3 (Yan17), in 2017, were both grown at the Managed Environment Facility (MEF) [34], located at Yanco Agricultural Institute (34.62°S, 146.43°E, elevation 164 m) in Southeastern Australia. All experiments were sown following canola or field pea break-crops and then managed with adequate nutrition and chemical controls as required for pest, weed, and leaf diseases.

Daily meteorological data was obtained from the Bureau of Meteorology (http://www.bom.gov.au/) weather station located closest to each experiment: Canberra Airport (station number 070351) and Yanco Agricultural Institute (station number 074037) (Table S1).

#### 2.1.1. Experiment 1 (GES15)

The soil at GES is classified as a deep yellow-red Podzolic with a fine sandy loam in the A horizon, changing abruptly at 0.30-0.50 m to a medium to hard clay texture in the B horizon [35]. The GES15 experiment, reported previously [17], comprised 13 contemporary bread wheat (*Triticum aestivum L.*) and two triticale (*x Triticosecale*) varieties chosen to represent the range in canopy architecture likely to be found within Australian commercial breeding programs and known to vary in flowering time by *ca.* 5 days from previous experiments at Yanco. The experimental plots were 15 m long with 10 rows spaced 0.18 m apart (orientated north-south) and paths between plots of *ca.* 0.4 m. The sowing density was 250 seeds/m^2^, and for five of the varieties known for erect canopy architecture, an additional low-density treatment (125 seeds/m^2^) was included to increase the range of above-ground biomass. The varieties were sown on 12 June 2015 into a randomized complete block design comprising 60 experimental plots (three replicates of 15 varieties sown at 250 seeds/m^2^ and five varieties sown at 125 seeds/m^2^).

#### 2.1.2. Experiments 2 (Yan16) and 3 (Yan17)

The soil at the Yanco MEF is classified as chromosol and has a clay-loam texture [36]. The Yan16 experiment was sown on 23 May in 2016 and Yan17 was sown 29 May in 2017. The Yan16 and Yan17 experiments comprised 240 and 64 experimental plots, respectively, 6 m long containing seven rows of 25 cm spacing (orientated north-south), sowing density of 200 seeds per m^2^ and paths between plots of *ca.* 0.4 m. The germplasm in Yan16 and Yan17 represented a series of near-isogenic wheat lines varying for a range of agronomic traits including plant height, tiller number, plant development, and canopy erectness. In Yan16, 98 genotypes were sown into a partial-replicate design experiment with the genotype replication averaging 2.45 and ranging from one to four. In Yan17, 41 genotypes (i.e., 41 of the 98 genotypes from Yan16) were sown into a partial-replicate design experiment with the genotype replication averaging 1.6 and ranging from one to two. Due to the limited rainfall in 2017 for the Yan17 experiment, sprinkler irrigation was applied on several separate days throughout the season, with amounts ranging from 15 to 37 mm (Table S1).

### 2.2. Phenotyping

#### 2.2.1. Above-Ground Biomass

The above-ground biomass (AGB) was determined from culms cut at ground level, always from the central rows of the plot and with at least one border row either side. The area sampled varied for each experiment as follows. GES15: 6rows × 0.18mrowspacing × 1.0mlength = 1.08m^2^. Yan16: 4rows × 0.25mrowspacing × 0.3mlength = 0.3m^2^. Yan17: 5rows × 0.25mrowspacing154 × 0.6mlength = 0.75m^2^. Throughout, a single area was sampled on each sampling date. Phenological growth stage (GS) was recorded at each AGB sampling event using the scale of [37]. For GES15, AGB was sampled on the following five dates with the average growth stage (GS) for all the plots shown in parentheses: 24-Sep-2015 (stem elongation, GS31); 7-Oct-2015 (stem elongation, GS32); 14-Oct-2015 (booting, GS41); 23-Oct-2015 (heading, GS55); and 30-Oct-2015 (anthesis, GS65). For Yan16, AGB was sampled at stem elongation (GS31), on 8-Aug-2016, and at anthesis (GS65) of each line which for 90% of the lines ranged from 22-Sep-2016 to 13-Oct-2016 (median anthesis date was 28-Sep-2016). For AGB sampled at anthesis, lines were sampled on the actual date they reached anthesis (or within two days of), and therefore, the lines were sampled on different dates. Samplings at stem elongation occurred for all lines on the same date (as indicated). In addition, AGB was sampled from a subset of 60 plots from within the Yan16 experiment whereby the genotypes were randomly sampled and unreplicated (i.e., 60 genotypes were represented). The additional AGB sampling from the subset of 60 plots is denoted Yan16sub. The AGB sampling size for Yan16sub was 0.75 m^2^ to enable more reliable comparison between AGB and the LiDAR. The Yan16sub sampling occurred on the following four dates with the average growth stage (GS) for the plots shown in parentheses: 22-Aug-2016 (stem elongation, GS35); 5-Sep-2016 (booting, GS45); 15-Sep-2016 (heading, GS55); and 25-Sep-2016 (early anthesis, GS61). For Yan17, AGB was sampled on the following four dates with the average growth stage (GS) for all the plots shown in parentheses: 28-Aug-2017 (stem elongation, GS35); 11-Sep-2017 (booting, GS45); 25-Sep-2017 (heading, GS55); and 9-Oct-2017 (anthesis, GS65).

The dry weight was determined from the AGB samples after drying at 70°C until reaching a constant dry weight. Leaf area (LI-COR LI-3000 conveyor belt system, Lincoln, Nebraska, USA) was determined from GES15, Yan16sub, and Yan17 AGB sampling events and expressed as leaf area per unit of land area (leaf area index, LAI). In addition, for Yan16sub and Yan17 AGB sampling events, the projected area of stems and ears was determined, summed with the leaf area and expressed as green area per unit of land area (green area index, GAI).

#### 2.2.2. Phenomobile Lite

The experiments were traversed with the previously described Phenomobile Lite system [17] on the day prior to an AGB sampling event (Figure S1). All genotypes in a given experiment were sampled on the same day. Traversing a given experiment with the Phenomobile Lite took approximately 20 minutes and typically occurred around solar noon.

The Phenomobile Lite is a portable buggy, powered by an electric wheel and steered by an operator walking behind, comprising a lightweight extruded aluminium frame with three wheels and a high-frequency laser scanner or LiDAR (SICK LMS400, Waldkirch, Germany, for which the technical specifications are as follows: 70° field of view; monochromatic laser 650 nm, 4.0-7.5 mW; 0.7-3.0 m range; 1 mm distance resolution; scanning and angular resolution of 250 Hz, 0.1°, 500 Hz, 0.25°). The LiDAR was mounted approximately 2.2 m above the ground. For the Yan16 and Yan17 experiments, a GreenSeeker sensor (Trimble, USA) was mounted on the Phenomobile Lite thereby enabling concurrent measurements of normalized difference vegetation index (NDVI) and LiDAR. The data streams were geocoded by means of a wheel encoder (submillimeter linear resolution) and GPS/IMU system (0.2° and <1.0 m accuracy) that were fitted to the Phenomobile Lite. All data were captured on a tablet (Panasonic F7-G1 Toughpad 10.1 inch HD daylight readable display with powered docking station, Microsoft Windows) for later processing.

#### 2.2.3. LiDAR Data Processing

As previously described [17], the LiDAR data was processed using a custom processing pipeline developed with Python 2.7 (Python Software Foundation, https://www.python.org/) whereby the LiDAR data was first geocoded with the wheel encoder and GPS/IMU data. The LiDAR data was then manually segmented into experimental plots through a custom-developed web interface according to the following:

- For GES15, Yan16sub, and Yan17, the section of the plot was selected from the same area where the AGB sampling was going to be performed (ca. 1.0 m^2^, 0.75 m^2^, and 0.75 m^2^, respectively)
- For Yan16, the central rows (i.e., excluding border rows) of each plot were selected to maximise the area sampled whilst avoiding sections of the plot previously sampled for AGB (equating to an area of at least 1.0 m^2^)

Previously described algorithms [16, 17] were then used to extract the following traits from the LiDAR data: crop height, 3D voxel index (3DVI), and 3D profile index (3DPI). The latter two, designed for the estimation of AGB from LiDAR, were highly correlated with destructive AGB in a prior study [17]. Briefly, 3DVI was calculated as the sum of the number of 0.05 m voxels [after 17] containing a LiDAR point, normalized by the total number of voxels (i.e., a voxel-based method). The 3DPI was calculated as the sum of the fractional number of points intercepted by the canopy in 0.01 m segments taken from the ground to the maximum crop height (i.e., a profile-based method). Crop height was obtained from the mean of the top 95^th^ percentile of the LiDAR height distribution for a given experimental plot, minus the height of the ground obtained from the average of the LiDAR returns from the soil, whereby the latter were determined for a given column of plots as the mode of heights in the point cloud (refer to Figure 4A in 17).

#### 2.2.4. Determination of Crop Growth Rate

Crop growth rate (CGR) between stem elongation and anthesis was calculated as the difference in AGB divided by the duration of each period for each genotype (i.e., *t* · *ha*^−1^ · day^−1^). CGR was calculated in the same way for the LiDAR biomass indices, 3DVI and 3DPI. As described above for the Yan16 experiment, AGB at GS65 was sampled on the actual date the entries reached anthesis (or within two days of), and therefore, the lines were sampled on different dates. Correspondingly, the LiDAR biomass indices, 3DVI and 3DPI, were linearly interpolated between individual sampling events (i.e., 15-Sep, 25-Sep, 21-Oct, and 25-Oct) for the GS65 date of each entry. The interpolated values were then used to determine CGR from the LiDAR biomass indices.

### 2.3. Statistical Analysis

The AGB, LAI, GAI, CGR, height, 3DVI, 3DPI, and NDVI data were analyzed after first checking for residual normality and error variance homogeneity at each date-by-time sampling event. For each trait, each event was analyzed separately using the SpATS package [38] (available from CRAN: http://cran.r-project.org/package=SpATS) in the R programming language (http://www.r-project.org/). Spatial effects were modelled on a row and column basis by specifying the P-spline ANOVA (PSANOVA) algorithm, with the number of segments set to the respective number of rows and columns from the experimental design. The following factors were modelled as random effects: genotype, row, and column. Repeatability (*ρ*), sometimes called broad-sense heritability [39–41], was then estimated using relevant variance components, namely, *ρ* = *σ*^2^*g*/(*σ*^2^*g* + (*σ*^2^∈/nrep)), where *σ*^2^_*g*_ and *σ*^2^∈ are the genotypic and residual variances, respectively, and nrep is the number of genotype replicates in the experiment. The best linear unbiased predictors of genotype effects (BLUPs) were predicted from a fitted SpATS object. Phenotypic correlations were estimated between BLUPs using Pearson correlation analysis with the pandas module in Python 3.5 and statistically significant associations denoted: ^∗∗∗∗^*P* < 0.0001; ^∗∗∗^*P* < 0.001; ^∗∗^*P* < 0.01; ^∗^*P* < 0.05. The SciPy module [42] in Python 3.5 was used to estimate linear least-squares regression between AGB and the two LiDAR biomass indices (raw data, i.e., nonspatially corrected, plot-level data) at individual sampling events where large biomass samples were taken (i.e., GES15, Yan16sub, and Yan17).

## 3. Results

### 3.1. Growing Conditions and Summary of Data

The meteorological conditions during the growing season for each experiment are summarised in Table S1. The GES15 and Yan16 experiments had excellent conditions for growth as related to the balance between reference evapotranspiration and water supply; in contrast, the limited water supply for Yan17 was inadequate for good growth. The results from the experiments are summarised for each measurement event in Tables S2, S3, and S4 for GES15, Yan16, and Yan17, respectively. Raw data (i.e., nonspatially corrected, plot-level data) are summarised as mean, standard deviation, and coefficient of variation for each phenotype. The mean CGRs were 0.16 (GES15), 0.14 (Yan16), and 0.12 (Yan17) (*t* · *ha*^−1^ · day^−1^) and within the range of that previously reported [4]. The higher mean CGRs attained for the GES15 and Yan16 experiments are consistent with their more favourable growing conditions, owing to greater water supply.

### 3.2. Phenotypic Correlations at Individual Sampling Events

**Figure 1:**
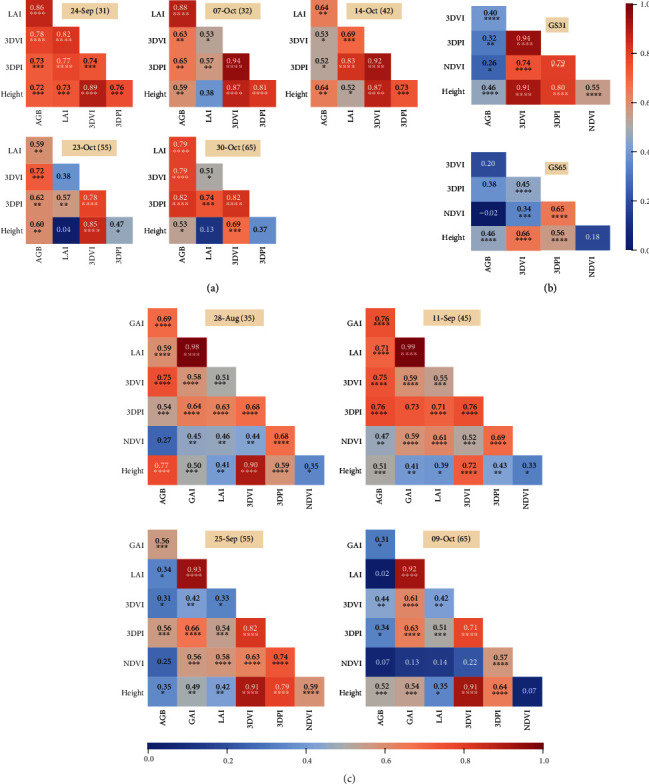
Phenotypic correlations, at individual sampling events, of the best linear unbiased predictors of genotype effects (BLUPs) for the three experiments: (a) GES15, (b) Yan16, and (c) Yan17. AGB: above-ground biomass; GAI: green area index; LAI: leaf area index; LiDAR biomass indices (3D vegetation index (3DVI) and 3D profile index (3DPI)); NDVI: normalized difference vegetation index; LiDAR crop height. For (a) GES15 and (c) Yan17, the sampling date is indicated and the phenological growth stage is shown in parentheses. For (b) Yan16, the phenological growth stage (GS) is indicated, and for AGB sampled at GS65, entries were sampled on the actual date they reached anthesis (or within two days of); therefore, the lines were sampled on different dates. 3DVI, 3DPI, NDVI, and height were interpolated between individual sampling events for the GS65 date of each entry.

The LiDAR biomass indices, 3DVI and 3DPI, were highly correlated with destructive AGB for the majority of discrete sampling events in the GES15 and Yan17 experiments, where large biomass samples were taken (1.08 and 0.75 m^2^, respectively). For GES15, the correlations between BLUPs ranged from 0.52 (*P* < 0.05) to 0.86 (*P* < 0.0001) (Figure 1(a)). For Yan16, where LiDAR biomass indices were compared to a more conventional AGB sample size (0.3 m^2^), correlations were not strong and ranged from 0.2 to 0.38 (*P* < 0.001) (Figure 1(b)). For Yan17, the correlations ranged from 0.31 (*P* < 0.05) to 0.76 (*P* < 0.0001) (Figure 1(c)). Correlations between NDVI and AGB for the Yan16 experiment were poor, ranging from -0.02 to 0.26 (*P* < 0.05). Correlations between NDVI and AGB ranged between 0.07 and 0.47 (*P* < 0.01) for the Yan17 experiment.

The correlations at individual sampling events, from 60 plots within the Yan16 experiment (Yan16sub), between AGB and the LiDAR biomass index, 3DPI, were high, ranging from 0.79 (*P* < 0.0001) to 0.85 (*P* < 0.0001) (Figure S2). The correlations between AGB and 3DVI were not as high, ranging from 0.51 (*P* < 0.0001) to 0.75 (*P* < 0.0001). Correlations between NDVI and AGB ranged from 0.41 (*P* < 0.01) to 0.66 (*P* < 0.0001).

For GES15, height was highly associated with both LiDAR indices until 14 Oct (GS42), ranging from 0.73 (*P* < 0.001) to 0.89 (*P* < 0.0001) (Figure 1(a)). Thereafter, the association was higher between height and 3DVI, than between height and 3DPI. Prior to 23-Oct in the GES15 experiment, the associations between LAI and both LiDAR indices were similar for a given event, ranging from 0.53 (*P* < 0.05) to 0.83 (*P* < 0.0001). However, from 23-Oct (GS55) onwards, the association between LAI and 3DPI was greater than the association between LAI and 3DVI. In summary, from 23-Oct (GS55) onwards in the GES15 experiment, LAI was more strongly associated with 3DPI than 3DVI and height was more strongly associated with 3DVI than 3DPI.

For Yan16, the association between height and both LiDAR indices was similar and high for each event, ranging from 0.56 (*P* < 0.0001) to 0.91 (*P* < 0.0001) (Figure 1(b)).

For Yan17, height was more strongly associated with 3DVI than 3DPI for all sampling events. Conversely, GAI and LAI were more strongly associated with 3DPI than 3DVI for all sampling events (Figure 1(c)).

For Yan16sub, GAI and LAI were more strongly associated with 3DPI than 3DVI for all sampling events (Figure S2). The association between height and both LiDAR indices was similarly high for each event, ranging from 0.59 (*P* < 0.0001) to 0.81 (*P* < 0.0001).

Intraclass correlations (ICCs), defined here as the correlation between sampling events for a given phenotype, followed a similar pattern across experiments and phenotypes: typically highly significant for consecutive events and of decreasing strength as the time between events increased (refer to Figures S3, S4, and S5 for GES15, Yan16, and Yan17, respectively). ICCs were notably high for 3DPI in the Yan16 experiment, ranging from 0.46 (*P* < 0.0001) to 0.99 (*P* < 0.0001). Conversely, the ICC was abnormally low between Yan17 events on 11-Sep and 17-Sep for 3DVI and 3DPI (0.00 and 0.02, respectively).

### 3.3. Linear Regression at Individual Sampling Events between LiDAR and AGB

Linear regression analysis for raw data (i.e., nonspatially corrected, plot-level data) at individual sampling events, where large biomass samples were taken, between AGB and the two LiDAR biomass indices is shown for GES15, Yan16sub, and Yan17 (Figure S6). Across the three experiments, the slopes between 3DVI and AGB ranged from 0.33 to 0.85 (mean 0.51) and the intercepts ranged from -0.96 to 2.79 (mean 0.97). For 3DPI, across the three experiments, the slopes ranged from 1.42 to 4.42 (mean 2.26) and the intercept ranged from 1.13 to 4.18 (mean 2.58). With the exception of 3DVI in GES15, the intercepts generally increased chronologically with the sampling events.

### 3.4. Phenotypic Repeatability Estimates at Individual Sampling Events

**Figure 2:**
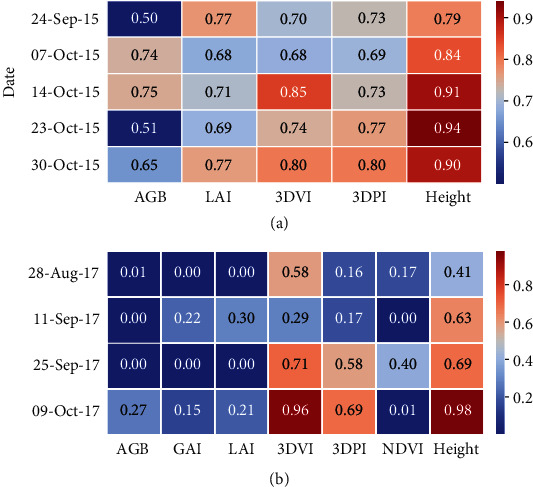
Repeatability estimates from the (a) GES15 and (b) Yan17 experiments for individual sampling events of above-ground biomass (AGB), green area index (GAI) (Yan17 only), leaf area index (LAI), the two LiDAR biomass indices (3D vegetation index (3DVI) and 3D profile index (3DPI)), normalized difference vegetation index (NDVI) (Yan17 only), and crop height derived from the LiDAR.

For GES15, repeatability estimates for the LiDAR biomass indices were at least equivalent to, and for the majority of events greater than, those for AGB (Figure 2(a)). For Yan16 at GS31, repeatability estimates for the LiDAR biomass indices and NDVI (0.88, 0.9, and 0.85 for 3DVI, 3DPI, and NDVI, respectively) were considerably greater than those for AGB (0.36) (Table 1). Repeatability estimates at GS65 were higher for the LiDAR biomass indices (0.82 and 0.84 for 3DVI and 3DPI, respectively) and NDVI (0.83) than AGB (0.6). For Yan17, repeatability estimates for the LiDAR indices were always greater than those for AGB, which was zero for all events except the final event on 9-Oct (Figure 2(b)). For NDVI, repeatability estimates were zero on two out of the four sampling events and always less than the LiDAR indices.

### 3.5. Analysis of Crop Growth Rate

For the GES15 experiment, the correlations between AGB CGR and CGR derived from the LiDAR biomass indices were 0.6 (*P* < 0.001) and 0.76 (*P* < 0.0001), for 3DVI and 3DPI, respectively (Figure S7(a)). The CGR correlations were not as strong for the Yan16 experiment: 0.12 and 0.38 (*P* < 0.0001), for 3DVI and 3DPI, respectively (Figure S7(b)). For the Yan17 experiment, the CGR correlations between AGB and the LiDAR indices were 0.39 (*P* < 0.05) and 0.45 (*P* < 0.001), for 3DVI and 3DPI, respectively (Figure S7(c)). Across all three experiments, the correlation between 3DPI CGR and AGB CGR was greater than that for 3DVI.

The repeatability estimates of CGR for AGB and the two LiDAR indices are shown in Table 2. For GES15, repeatability estimates of CGR were appreciably greater for 3DVI (0.57) and 3DPI (0.78) than for AGB (0.45). Similarly, for the larger experiment, Yan16, repeatability estimates of CGR for 3DVI (0.78) and 3DPI (0.81) were more than twofold than those of AGB (0.31). For the Yan17 experiment, the repeatability estimate of CGR for 3DVI (0.93) was more than twofold than that of 3DPI (0.34) and AGB (0.39).

## 4. Discussion

Across three experiments, contrasting in available water supply and comprising up to 98 wheat genotypes varying for canopy architecture, concurrent measurements of LiDAR and destructive AGB were made at several stages of development from jointing to anthesis. The consistently high correlations between the large biomass samples and the LiDAR biomass indices provide confidence in the LiDAR indices as effective surrogates for destructive AGB. These results accord with a recent study comprising eight bread-wheat cultivars grown across eight sites, where LiDAR-derived crop-volume estimates were often significantly correlated with AGB and broad-sense heritability estimates from LiDAR were typically greater than those from AGB [32]. In our experiments, the repeatability of the LiDAR biomass indices at discrete events was at least similar to and often higher than AGB, particularly under water limitation. The correlations between calculated CGR for AGB and the LiDAR indices were moderate to high and varied between experiments (Figure S7). However, across all experiments, the repeatability of the CGR derived from the LiDAR indices was appreciably greater than that for AGB (Table 2), with the exception of the 3DPI in the water-limited environment of the Yan17 experiment. Measurements of AGB and CGR are laborious and time-consuming and the repeatability is often low, more so on large experiments where it can take several days for a team of workers to complete the process of AGB sampling, drying, and weighing. In contrast, the process of traversing an experiment with the Phenomobile Lite and processing the data to derive the LiDAR biomass indices, NDVI, and crop height for every plot can take as little as one hour for an experiment comprising 250 plots.

### 4.1. High Repeatability Estimates from LiDAR Biomass Indices

Both LiDAR biomass indices were highly repeatable from stem elongation growth stage to anthesis, as shown through the consistently high repeatability estimates (Figure 2 and Table 1) and intraclass correlations between consecutive events (Figures S3, S4, and S5). The repeatability estimates for AGB were highest in GES15. This is probably due to the sample size of 1.08 m^2^, a larger sample size than typically used in prebreeding studies. For example, a quadrat sample of 0.25 m^2^ was described by [14] and a similar quadrat size of 0.22 to 0.36 m^2^ was described by [15]. In the Yan16 experiment, the small quadrat size of *ca.* 0.25 m^2^ for AGB resulted in low and moderate repeatability estimates at GS31 (0.36) and GS65 (0.6) (Table 1), respectively. These values are similar to previously reported repeatability estimates for biomass from a sample area of 0.4 m^2^ [43] under fully irrigated conditions, which ranged from 0.24 to 0.5 (measured 40 days after emergence and at anthesis plus seven days, respectively). Under water limitation in the Yan17 experiment described herein, despite using a large sample size (0.75 m^2^), the repeatability estimates for AGB were low, ranging from 0.0 to 0.27 (Figure 2(b)).

The repeatability estimates reported herein for the LiDAR biomass indexes are similar in range to the predicted AGB heritabilities reported by [44] (0.78 to 0.84) for an experiment comprising 647 doubled haploid triticale lines derived from four families. In the study of [44], AGB was estimated using the “Breed Vision” platform [45] comprising multiple optical sensors including three laser distance sensors, two 3D-Time-of-Flight cameras, a hyperspectral imaging system, and light curtains, used to avoid influence of direct solar radiation. A multiple linear regression model was used to “fuse” the sensor data and generate a biomass prediction model. Similarly, Walter et al. [32] reported heritabilities for LiDAR-derived crop volume estimates ranging from 0.32 to 0.90. These heritabilities were typically greater than those for AGB, which ranged from 0.12 to 0.78. The high repeatability for nondestructive measures of AGB, reported in both the present study and [32, 44], demonstrates the utility of LiDAR-based platforms for potential use in plot-scale phenotyping within a genetics study or within a plant breeding program.

### 4.2. Analysis of the Difference between the LiDAR Biomass Indices: 3DVI and 3DPI

The LiDAR biomass indices represent two different approaches for estimating AGB from the LiDAR point cloud: a voxel-based method (3D voxel index (3DVI)) and a profile-based method (3D profile index (3DPI)) [see 17]. The association between height, LAI/GAI, and the respective LiDAR biomass indices provides a greater understanding of the differences between the LiDAR indices and their relative performance across the three experiments herein. In our experiments, the profile-based method, 3DPI, showed a greater dependence on LAI and GAI than 3DVI. Conversely, the voxel-based method, 3DVI, showed a greater dependence on height than 3DPI.

In Yan16, the growing conditions were conducive to high biomass and leaf area, and the 3DPI outperformed the 3DVI: the associations between AGB were greater for 3DPI than 3DVI (Figure S6) and the repeatability estimates were higher for 3DPI than 3DVI for both the discrete sampling events, with the exception of the final two events (Table 1), and CGR (Table 2). The superior performance of the 3DPI under favourable growth conditions may be due to saturation of the 3DVI under high biomass, whereby all the voxels fill up resulting in lower granularity. In contrast, under the water-limited conditions in the Yan17 experiment, 3DVI performed better than 3DPI in terms of repeatability estimates, which were greater for 3DVI than 3DPI for both the discrete sampling events (Figure 2(b)) and CGR (Table 2). However, the associations between AGB were appreciably greater for 3DVI than 3DPI for only two of the four sampling events (Figure 1(c)). Nevertheless, the 3DVI generally performed better than the 3DPI under the water-limited conditions in Yan17. This may be due to the 3DVI having a lower dependence on leaf area compared to the 3DPI.

Linear regression analysis of the raw data at individual sampling events between the large AGB samples and either 3DVI or 3DPI showed that although these variables were highly correlated, the regression parameters varied across sampling events. In particular, the intercept with AGB typically increased chronologically with the sampling events. Similarly, although Walter et al. [32] reported good associations between LiDAR-derived volume estimates of the crop canopy and AGB, the regression parameters appeared to vary across sampling events according to the scatter plots presented (refer to Figure 7 in [32], the regression parameters were not reported). Therefore, in the absence of stable intercepts and slopes between AGB and LiDAR across sampling events (reported herein and in [32]), one cannot expect a universal function to accurately predict AGB from LiDAR across multiple sites, even for one species (wheat). It is noted however that the LiDAR indices used herein and in [32] directly arise from LiDAR metrics applied to the LiDAR point cloud. An alternative approach involves the application of machine learning to directly classify plant organs and features within the canopy, including the ground [46]. The latter approach may improve the accuracy of nondestructive estimates of AGB and further work is required.

### 4.3. Application to Prebreeding Research and Breeding

This study demonstrates the utility of LiDAR mounted on the Phenomobile Lite as a tool for nondestructive, reliable, and repeatable quantification of both AGB and CGR from stem elongation to anthesis. This is particularly relevant to wheat improvement studies, where increased preanthesis CGR has been associated with genetic progress in wheat grain yield, establishment of grain number, and potential grain size [3, 7, 8]. The nondestructive LiDAR method described herein is ideally suited to screening large experiments for preanthesis CGR; a trait that is otherwise difficult to measure in large experiments.

LiDAR estimation of AGB and CGR could assist breeders with discarding poor performing lines. In early generations, breeders are not confident selecting the top 10% performing lines, so focus instead on discarding poor performers. An indirect estimate of poor performance using LiDAR could assist with discarding poor lines and thereby enable an increased selection intensity based on discarding a greater number of poor performers. However, there is evidence suggesting that a bordered plot is required for reliable estimates of growth-related traits [47], including CGR, to overcome the confounding effects of competition for resources (light, water, and nutrients) that are evident in the spaced rows or unbordered plots typically used in breeder's nurseries. There is the possibility, therefore, for selection in spaced rows based on CGR to favour more competitive lines. Further work is required to test that genotypic variation in CGR measured on the short rows typically used in breeder's nurseries is reliably expressed in full plots and not confounded by edge effects. Nevertheless, CGR estimates on bordered plots at latter stages of a breeding program, in addition to yield measurements, could improve genomic selection models and assist with selection of parents for crossing.

Plot size in breeder's trials is typically smaller than that used in this study (e.g., [47]), and based on a walking speed of 5 km/h, the Phenomobile Lite system presented herein could traverse *ca.* 2,500 plots of 4 m^2^ size per hour. Nevertheless, the Phenomobile Lite platform in its present form may not be amenable for use by breeders and several technical improvements to the Phenomobile Lite system could assist uptake by breeders, including mounting the LiDAR on a tractor [48, 49], using a smaller and lighter LiDAR on an unmanned aerial vehicle (UAV) and use of GPS georeferencing to increase data processing speed, and travelling at right angle to the direction of seeding in order to scan two plots simultaneously. The latter point was recently highlighted [49]: traversing the plots in the direction of seeding (in the case of the Phenomobile Lite) greatly increases the travel distance when compared to systems that travel at right angle to the direction of seeding and scan two plots simultaneously (refer to Figures 2 and 6 in 49). Although the former may scan the plot more completely than the latter, thereby resulting in data of superior quality, the difference in travel distance is significant (nearly eightfold in the example presented by 49).

## 5. Conclusions

Preanthesis above-ground biomass (AGB) and crop growth rate (CGR) have been associated with genotypic variation in wheat grain yield potential and therefore identified for phenotypic selection in genetics studies or in plant breeding trials. However, destructive sampling of these traits is laborious and often the measurement repeatability is low. Reliable, repeatable, and nondestructive methods for measuring AGB and CGR are required. LiDAR mounted on the Phenomobile Lite provides a repeatable, nondestructive, and high-throughput estimate of AGB and CGR. Herein, the use of the LiDAR indices as effective surrogates for AGB was supported by high phenotypic correlations at discrete sampling events between the AGB samples and the LiDAR-derived biomass indices. However, the regression parameters between AGB and the LiDAR indices varied across sampling events and at this stage are not sufficiently robust for universal predication of absolute AGB from LiDAR. Nevertheless, repeatability estimates from either LiDAR index were consistently higher than those from AGB, both at discrete time points and when CGR was calculated. This study provides promising support for the reliable use of ground-based LiDAR, as a surrogate measure of AGB and CGR, for screening germplasm in research and wheat breeding.

## Figures and Tables

**Table 1:** Repeatability estimates from the Yan16 experiment for above-ground biomass (AGB), the two LiDAR biomass indices (3D vegetation index (3DVI) and 3D profile index (3DPI)), normalized difference vegetation index (NDVI), and crop height derived from the LiDAR. The date of each sampling event is indicated as well as the dates of phenological growth stages (GS) 31 and 45 (as attained by 50% of entries). For 90% of the lines, GS65 ranged from 22-Sep to 13-Oct (median GS65 date was 28-Sep). For AGB sampled at GS65, entries were sampled on the actual date they reached anthesis (or within two days of); therefore, the lines were sampled on different dates and date is denoted “various.” Correspondingly, the values of 3DVI, 3DPI, NDVI, and height were interpolated between individual sampling events (i.e., 15-Sep, 25-Sep, 21-Oct, and 25-Oct) for the GS65 date of each entry.

Date	GS	AGB	3DVI	3DPI	NDVI	Height
8-Aug	31	0.36	0.88	0.90	0.85	0.89
16-Aug	—	—	0.78	0.87	0.83	0.81
22-Aug	—	—	0.68	0.79	0.61	0.84
6-Sep	45	—	0.69	0.82	0.62	0.90
15-Sep	—	—	0.70	0.78	0.39	0.90
25-Sep	—	—	0.77	0.79	0.54	0.92
Various	65	0.60	0.82	0.84	0.83	0.94
21-Oct	—	—	0.73	0.66	0.66	0.91
25-Oct	—	—	0.66	0.63	0.63	0.91

**Table 2:** Repeatability estimates for crop growth rate (CGR denoted *Δ*), between stem elongation (GS31) and anthesis (GS65), for above-ground biomass (AGB) and the two LiDAR biomass indices (3D vegetation index (3DVI) and 3D profile index (3DPI)). The three experiments are denoted GES15, Yan16, and Yan17.

Experiment	*Δ* AGB	*Δ* 3DVI	*Δ* 3DPI
GES15	0.45	0.57	0.78
Yan16	0.31	0.78	0.81
Yan17	0.39	0.93	0.34

## Supplementary Materials

Supplementary Materials Figure S1: Phenomobile Lite comprising LiDAR, RGB camera, GreenSeeker R, and tablet mounted on an aluminium frame with adjustable wheel spacing to accommodate different plot widths (1.75-2.2 m) and ground clearance of canopy heights up to 1.5 m. Figure S2: correlations, at individual sampling events, from 60 plots within the Yan16 experiment (denoted Yan16sub) between above-ground biomass (AGB), green area index (GAI), leaf area index (LAI), the two LiDAR biomass indices (3D vegetation index (3DVI) and 3D profile index (3DPI)), normalized difference vegetation index (NDVI), and crop height derived from the LiDAR. Figure S3: intraclass correlations (i.e., between sampling events) of the best linear unbiased predictors of genotype effects (BLUPs) for the GES15 experiment between above-ground biomass (AGB), the two LiDAR biomass indices (3D vegetation index (3DVI) and 3D profile index (3DPI)), and crop height derived from the LiDAR. The date of each sampling event is indicated. Figure S4: intraclass correlations (i.e., between sampling events) of the best linear unbiased predictors of genotype effects (BLUPs) for the Yan16 experiment between the two LiDAR biomass indices (3D vegetation index (3DVI) and 3D profile index (3DPI)), normalized difference vegetation index (NDVI), and crop height derived from the LiDAR. The date of each sampling event is indicated. Figure S5: intraclass correlations (i.e., between sampling events) of the best linear unbiased predictors of genotype effects (BLUPs) for the Yan17 experiment between the two LiDAR biomass indices (3D vegetation index (3DVI) and 3D profile index (3DPI)), normalized difference vegetation index (NDVI), and crop height derived from the LiDAR. The date of each sampling event is indicated. Figure S6: linear regression analysis of nonspatially corrected, plot-level data at individual sampling events between above-ground biomass (AGB) and the two LiDAR biomass indices: 3D vegetation index (3DVI, left panels) and 3D profile index (3DPI, right panels). Figure S7: phenotypic correlations of the best linear unbiased predictors of genotype effects (BLUPs) for crop growth rate (CGR denoted *Δ*) from above-ground biomass (AGB), LiDAR 3D vegetation index (3DVI), and LiDAR 3D profile index (3DPI). CGR between stem elongation and anthesis was calculated as the difference in AGB divided by the duration of each period for each genotype. Table 1: meteorological conditions during the growing season for the three experiments, denoted GES15, Yan16, and Yan17. Table 2: summary of data from experiment. Mean (M), standard deviation (SD), and coefficient of variation (CV) for above-ground biomass (AGB), leaf area index (LAI), 3D vegetation index (3DVI), 3D profile index (3DPI), crop height, and crop growth rate (CGR). Table 3: summary of data from Yan16 experiment. Mean (M), standard deviation (SD), and coefficient of variation (CV) for above-ground biomass (AGB), 3D vegetation index (3DVI), 3D profile index (3DPI), normalized difference vegetation index (NDVI), crop height, and crop growth rate (CGR). Table 4: summary of data from Yan17 experiment. Mean (M), standard deviation (SD), and coefficient of variation (CV) for above-ground biomass (AGB), green area index (GAI), leaf area index (LAI), 3D vegetation index (3DVI), 3D profile index (3DPI), normalized difference vegetation index (NDVI), crop height, and crop growth rate (CGR).
