# Supplementary material for: Ground-Based LiDAR Improves Phenotypic Repeatability of Above-Ground Biomass and Crop Growth Rate in Wheat
Source: Plant Phenomics. 2020 May 26;2020:8329798. doi: 10.34133/2020/8329798 (PMC7706344; doi:10.34133/2020/8329798)
Supplement: Supplementary Materials — Figure S1: Phenomobile Lite comprising LiDAR, RGB camera, GreenSeeker R, and tablet mounted on an aluminium frame with adjustable wheel spacing to accommodate different plot widths (1.75-2.2 m) and ground clearance of canopy heights up to 1.5 m. Figure S2: correlations, at individual sampling events, from 60 plots within the Yan16 experiment (denoted Yan16sub) between above-ground biomass (AGB), green area index (GAI), leaf area index (LAI), the two LiDAR biomass indices (3D vegetation index (3DVI) and 3D profile index (3DPI)), normalized difference vegetation index (NDVI), and crop height derived from the LiDAR. Figure S3: intraclass correlations (i.e., between sampling events) of the best linear unbiased predictors of genotype effects (BLUPs) for the GES15 experiment between above-ground biomass (AGB), the two LiDAR biomass indices (3D vegetation index (3DVI) and 3D profile index (3DPI)), and crop height derived from the LiDAR. The date of each sampling event is indicated. Figure S4: intraclass correlations (i.e., between sampling events) of the best linear unbiased predictors of genotype effects (BLUPs) for the Yan16 experiment between the two LiDAR biomass indices (3D vegetation index (3DVI) and 3D profile index (3DPI)), normalized difference vegetation index (NDVI), and crop height derived from the LiDAR. The date of each sampling event is indicated. Figure S5: intraclass correlations (i.e., between sampling events) of the best linear unbiased predictors of genotype effects (BLUPs) for the Yan17 experiment between the two LiDAR biomass indices (3D vegetation index (3DVI) and 3D profile index (3DPI)), normalized difference vegetation index (NDVI), and crop height derived from the LiDAR. The date of each sampling event is indicated. Figure S6: linear regression analysis of nonspatially corrected, plot-level data at individual sampling events between above-ground biomass (AGB) and the two LiDAR biomass indices: 3D vegetation index (3DVI, left panels) and 3D profil [file 8329798.f1.zip › revised_supplementary_material_PlantPhenomics-D-19-00047.pdf]

Supplementary Material: Ground-based LiDAR improves phenotypic repeatability of above-ground biomass and crop growth rate in wheat

David M. Deery*, Greg J. Rebetzke, Jose A. Jimenez-Berni, Anthony G. Condon, David J. Smith, Kathryn M. Bechaz, William D. Bovill

*Correspondence:
Author Name: David M. Deery
david.deery@csiro.au

1 FIGURES

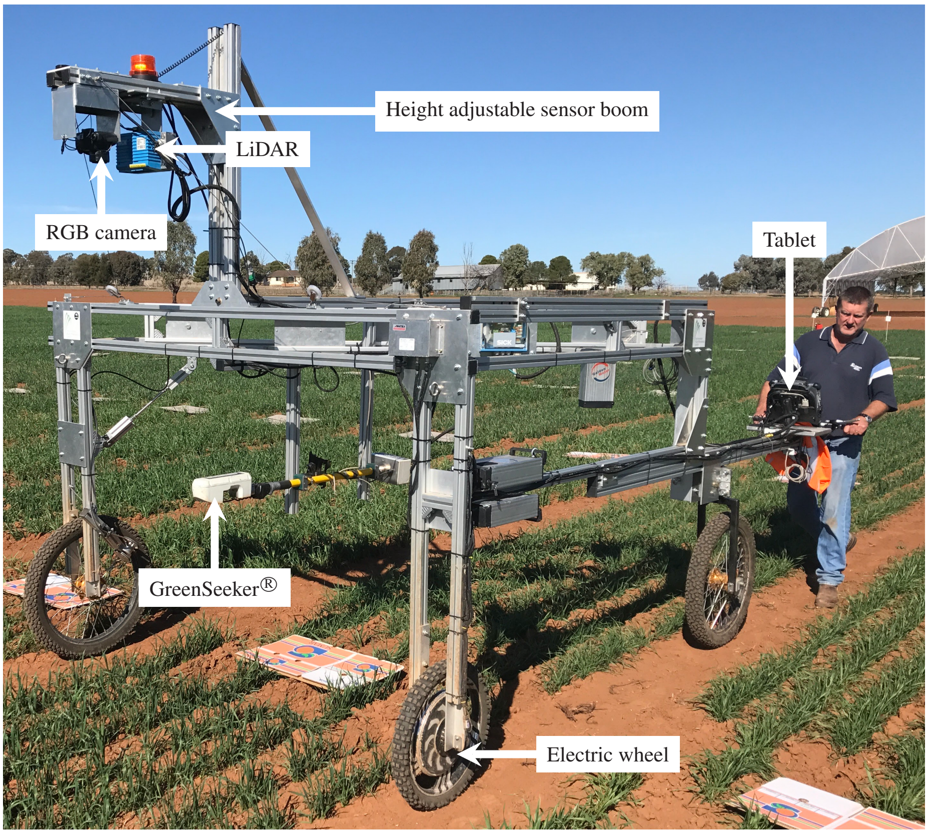

Figure S1. Phenomobile Lite comprising LiDAR, RGB camera, GreenSeeker® and tablet mounted on an aluminium frame with adjustable wheel spacing to accommodate different plot widths (1.75-2.2 m) and ground-clearance of canopy heights up to 1.5 m. The cardboard marks the transverse path across plot ends. The height adjustable sensor boom (2.0-2.5 m) enabled data capture from crop emergence to maturity. The Phenomobile Lite was powered by an electric wheel and steered by an operator walking behind. Data was captured on a tablet and processed through a web interface whereby the user processed the plot data in a semi-automated fashion.

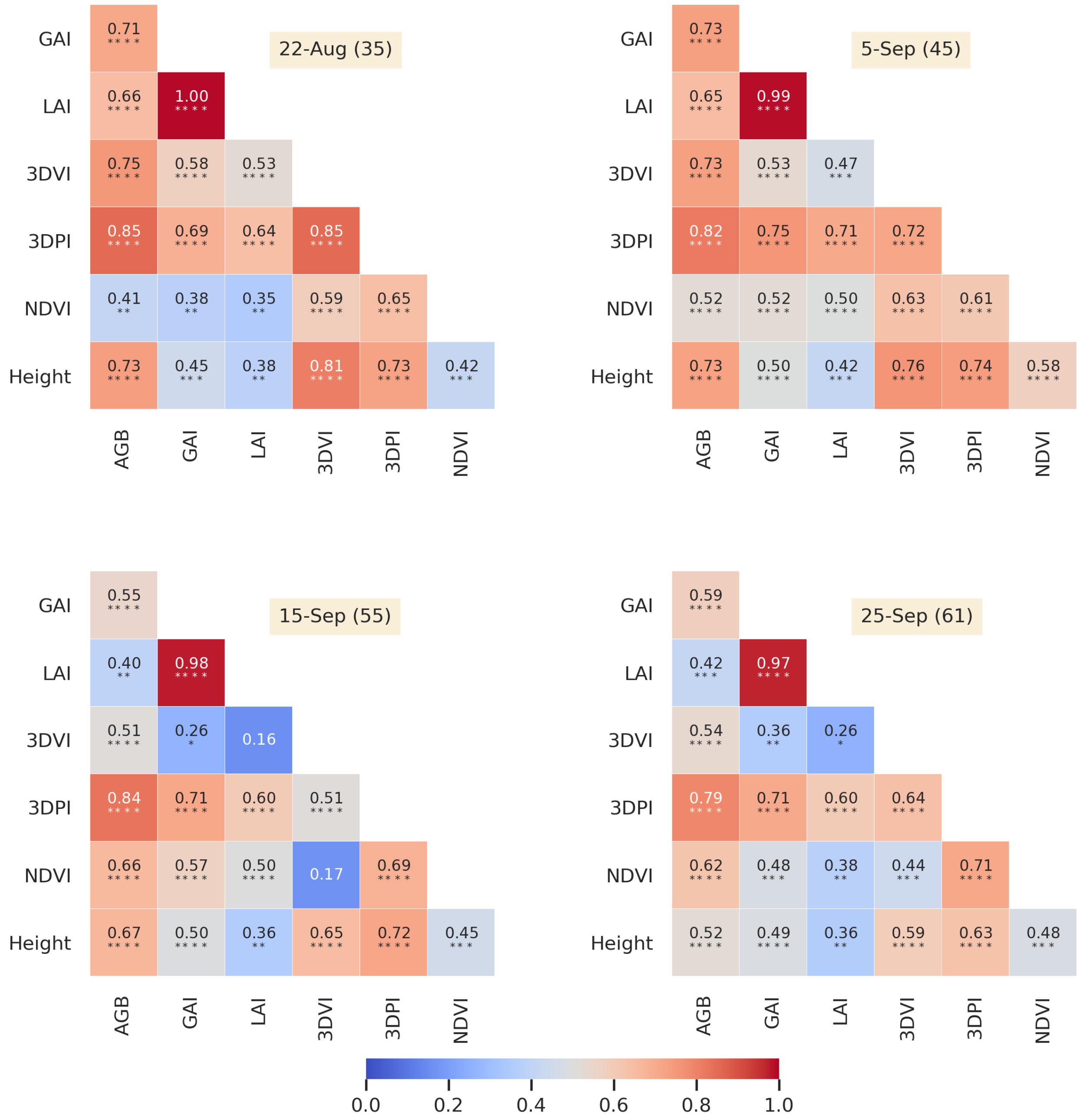

Figure S2. Correlations, at individual sampling events, from 60 plots within the Yan16 experiment (denoted Yan16sub) between: above-ground biomass (AGB); green area index (GAI); leaf area index (LAI); the two LiDAR biomass indices (3D vegetation index (3DVI) and 3D profile index (3DPI)); normalized difference vegetation index (NDVI); and crop height derived from the LiDAR. The date and average phenological growth stage (GS) for each sampling event is indicated. The genotypes were randomly sampled and unreplicated (i.e. 60 genotypes are represented). The AGB sample size was 0.75 m^2.

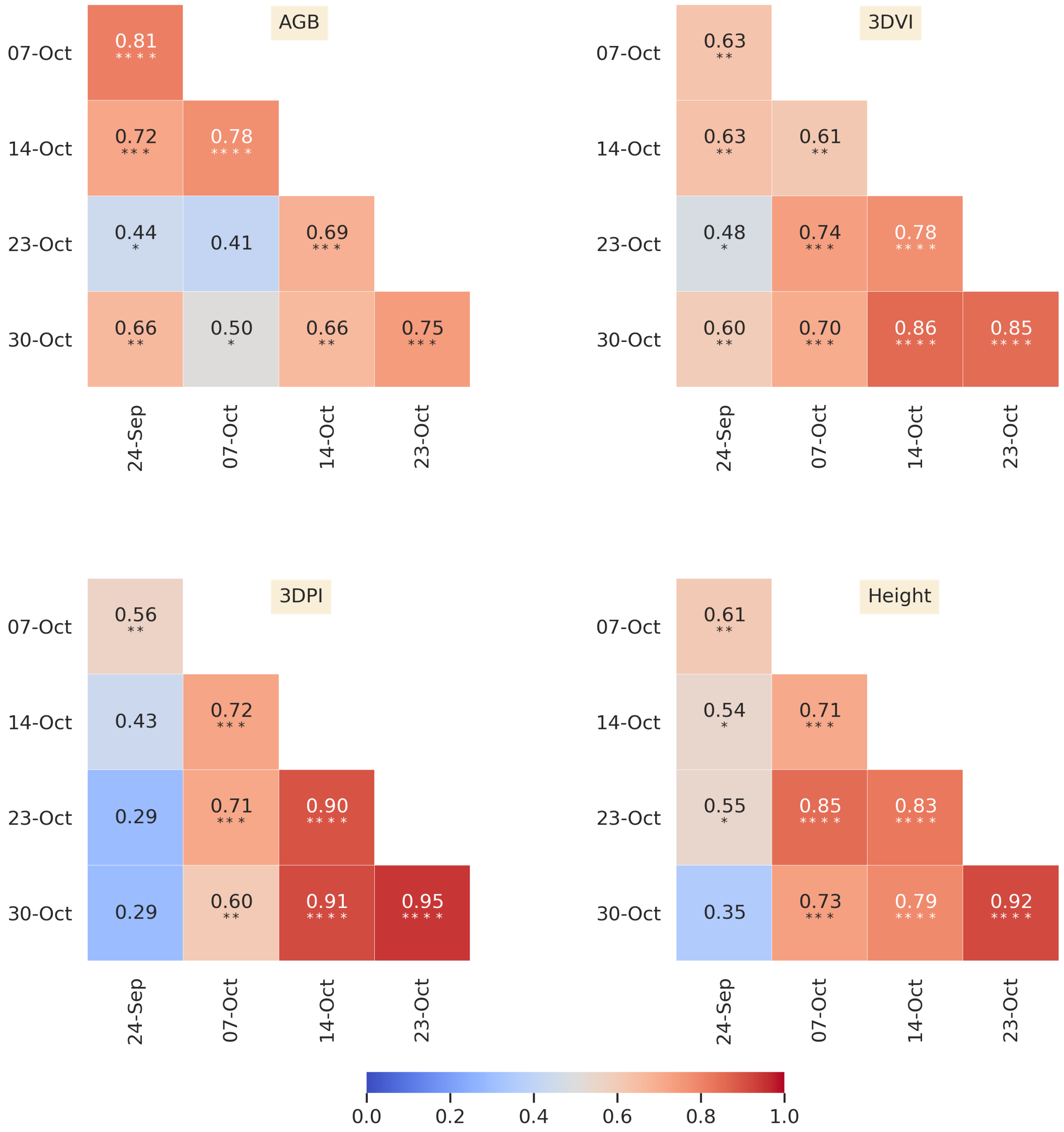

Figure S3. Intra-class correlations (i.e. between sampling events) of best linear unbiased predictors of genotype effects (BLUPs) for the GES15 experiment between: above-ground biomass (AGB); the two LiDAR biomass indices (3D vegetation index (3DVI) and 3D profile index (3DPI)); and crop height derived from the LiDAR. The date of each sampling event is indicated.

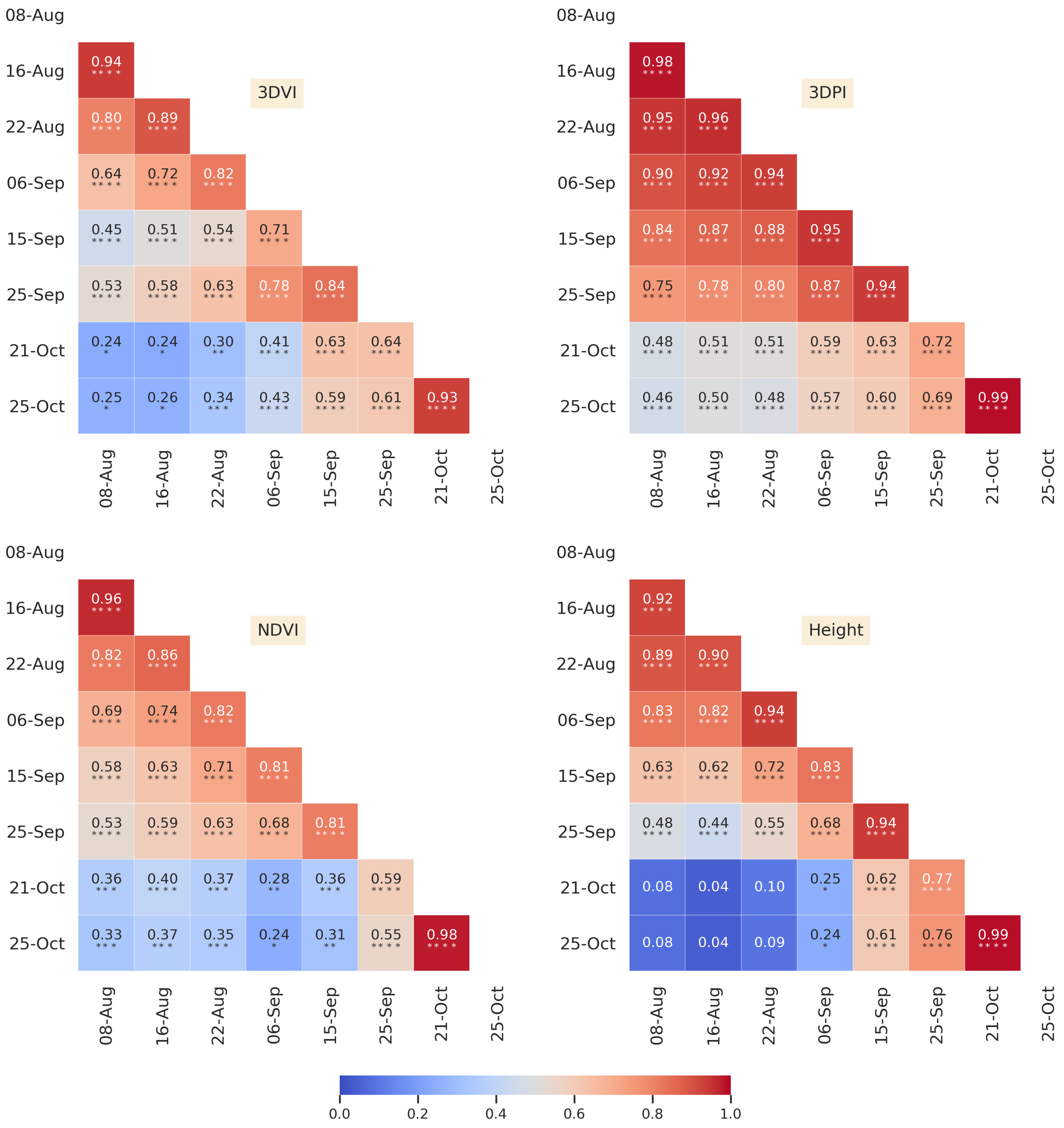

Figure S4. Intra-class correlations (i.e. between sampling events) of best linear unbiased predictors of genotype effects (BLUPs) for the Yan16 experiment between: the two LiDAR biomass indices (3D vegetation index (3DVI) and 3D profile index (3DPI)); normalized difference vegetation index (NDVI); and crop height derived from the LiDAR. The date of each sampling event is indicated.

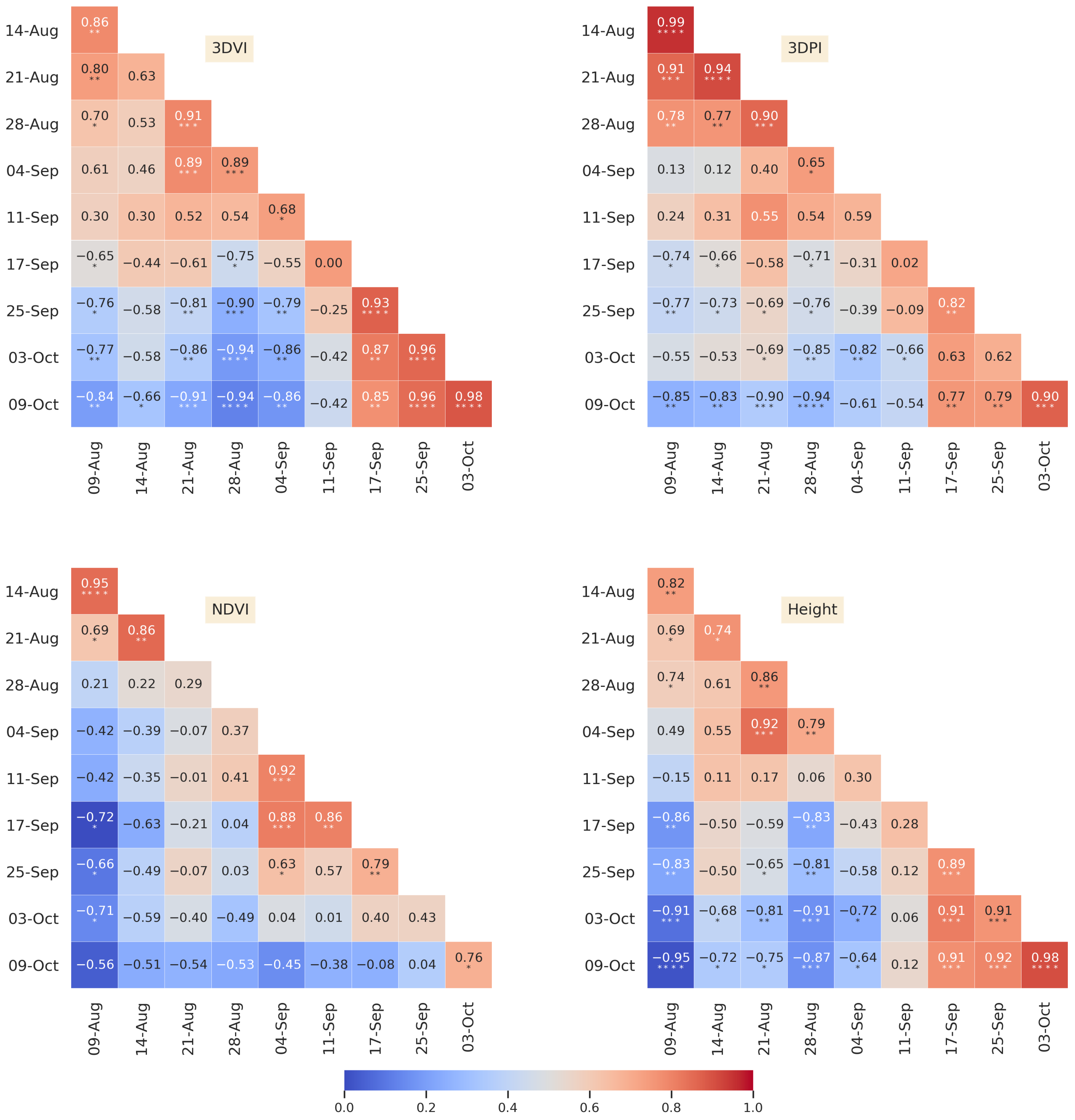

Figure S5. Intra-class correlations (i.e. between sampling events) of best linear unbiased predictors of genotype effects (BLUPs) for the Yan17 experiment between: the two LiDAR biomass indices (3D vegetation index (3DVI) and 3D profile index (3DPI)); normalized difference vegetation index (NDVI); and crop height derived from the LiDAR. The date of each sampling event is indicated.

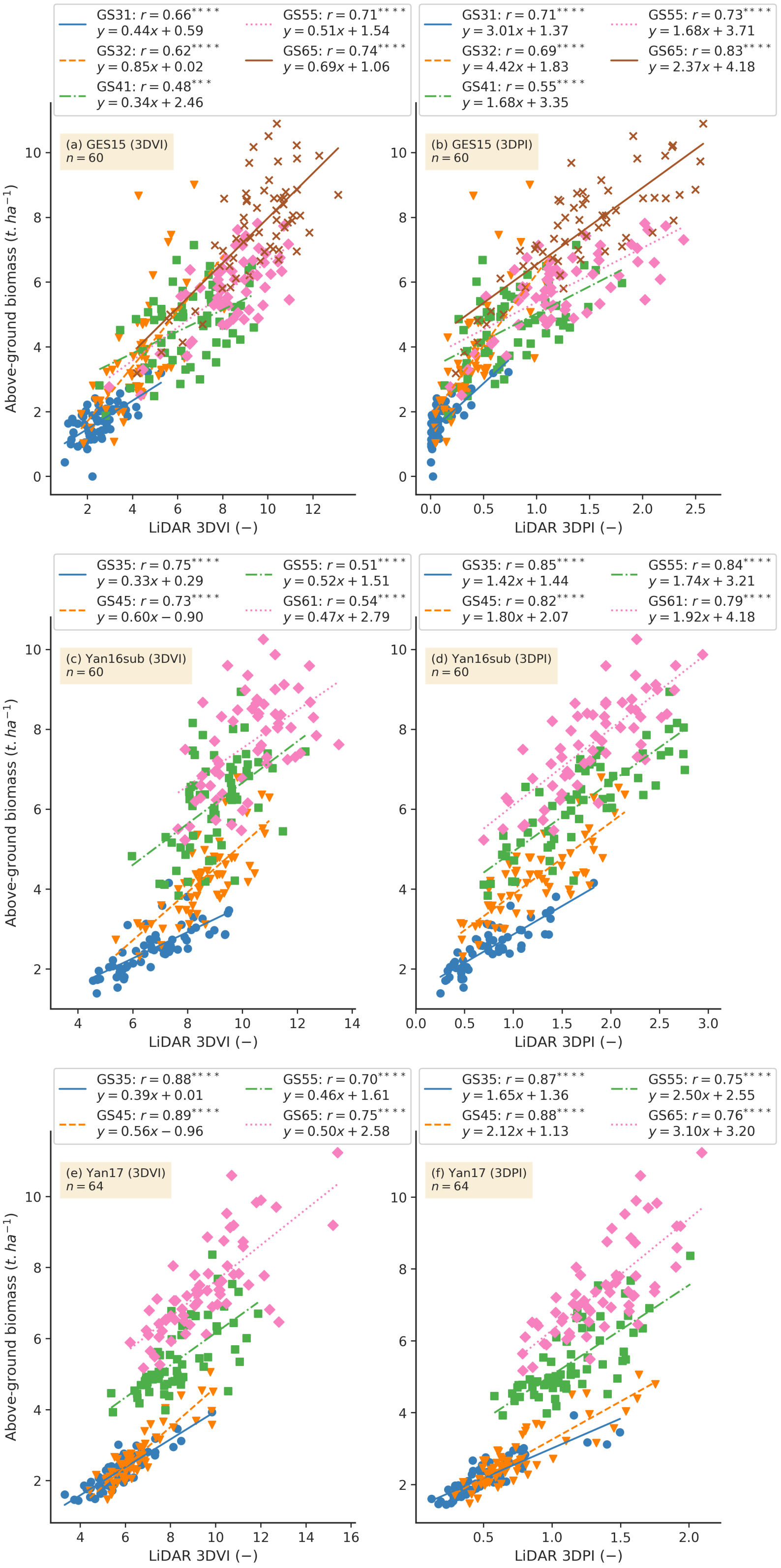

Figure S6. Linear regression analysis of non-spatially corrected, plot-level data at individual sampling events between above-ground biomass (AGB) and the two LiDAR biomass indices: 3D vegetation index (3DVI, left panels) and 3D profile index (3DPI, right panels). For each sampling event, the phenological growth stage (GS) is indicated with the Pearson correlation, r, and the fitted linear regression equation with statistically significant non-zero slopes denoted. The three experiments are indicated: GES15 ((a) and (b)); Yan16sub ((c) and (d)) (comprising 60 plots from within the Yan16 experiment); Yan17 ((e) and (f)).

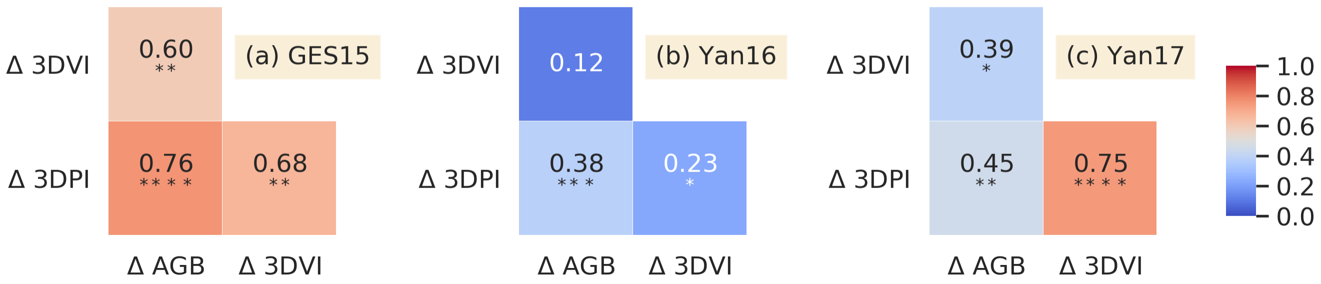

Figure S7. Phenotypic correlations of best linear unbiased predictors of genotype effects (BLUPs) for crop growth rate (CGR denoted Δ) from above-ground biomass (AGB), LiDAR 3D vegetation index (3DVI) and LiDAR 3D profile index (3DPI). CGR between stem elongation and anthesis was calculated as the difference in AGB divided by the duration of each period for each genotype. CGR was calculated in the same way for the LiDAR biomass indices, 3DVI and 3DPI.

2 TABLES

Table 1. Meteorological conditions during the growing season for the three experiments, denoted GES15, Yan16 and Yan17. Monthly means of daily accumulated solar radiation, minimum (T_{min}) and maximum air temperature (T_{max}). Rain, irrigation and reference evapotranspiration are monthly totals. Total irrigation and irrigation plus rain are shown in parenthesis.

Experiment	Month	Radiation ($MJ.m^{-2}$)	T_{min} (°C)	T_{max} (°C)	Rain (mm)	Irrigation (mm)	Evapotranspiration (mm)
GES15	Jun	8.6	-0.8	13.8	55	0	34
	Jul	8.7	-0.7	11.5	37	0	40
	Aug	11	1.2	13.7	67	0	54
	Sep	17.1	1.5	17.4	14	0	88
	Oct	20.2	8.3	24.7	27	0	131
	Nov	23.5	10.9	25.3	68	0	163
	Total	-	-	-	268	(0, 268)	510
Yan16	Jun	7.7	7.3	13.9	112	0	31
	Jul	8.4	6.7	14.2	58	0	36
	Aug	11.9	5.8	15.8	66	0	56
	Sep	14	8.6	17.5	138	0	67
	Oct	20.9	8.6	21.3	46	0	130
	Nov	25.1	12.4	28.1	35	0	188
	Total	-	-	-	455	(0, 455)	508
Yan17	Jun	9.7	2.7	15.9	2	0	37
	Jul	9.8	4.7	16	24	0	53
	Aug	12.8	4.2	15.9	33	0	62
	Sep	16.7	7.4	21.7	1	81	131
	Oct	21.8	11.6	26	28	102	163
	Nov	25.6	15.6	30.2	21	18	203
	Total	-	-	-	109	(201, 310)	649

Table 2. Summary of data from GES15 experiment. Mean (M), standard deviation (SD) and coefficient of variation (CV) for above-ground biomass (AGB), leaf area index (LAI), 3D vegetation index (3DVI), 3D profile index (3DPI), crop height and crop growth rate (CGR). The 3DVI, 3DPI and crop height were derived from the LiDAR. CGR between stem elongation and anthesis was calculated as the difference in AGB divided by the duration (i.e. $t.ha^{-1}.day^{-1}$). CGR was calculated in the same way for the LiDAR biomass indices, 3DVI and 3DPI. The phenological growth stage (GS) and date is indicated for each sampling event.

		24-Sep<br>31	7-Oct<br>32	14-Oct<br>42	23-Oct<br>55	30-Oct<br>65	CGR<br>31 to 65
AGB $(t.ha^{-1})$	M	1.69	3.64	4.64	5.79	7.59	0.16
	SD	0.58	1.62	1.15	1.16	1.66	0.04
	CV	0.34	0.45	0.25	0.20	0.22	0.24
LAI $(-)$	M	1.41	2.38	2.93	2.38	2.09	–
	SD	0.56	0.99	1.02	0.71	0.83	–
	CV	0.40	0.42	0.35	0.30	0.40	–
3DVI $(-)$	M	2.51	4.28	6.50	8.34	9.43	0.19
	SD	0.88	1.19	1.62	1.62	1.77	0.05
	CV	0.35	0.28	0.25	0.19	0.19	0.24
3DPI $(-)$	M	0.11	0.41	0.77	1.25	1.44	0.04
	SD	0.14	0.25	0.38	0.50	0.58	0.01
	CV	1.31	0.62	0.49	0.40	0.40	0.40
Height (m)	M	0.18	0.31	0.44	0.60	0.70	–
	SD	0.06	0.07	0.09	0.11	0.12	–
	CV	0.34	0.24	0.21	0.19	0.18	–

Table 3. Summary of data from Yan16 experiment. Mean (M), standard deviation (SD) and coefficient of variation (CV) for above-ground biomass (AGB), 3D vegetation index (3DVI), 3D profile index (3DPI), normalized difference vegetation index (NDVI), crop height and crop growth rate (CGR). The 3DVI, 3DPI and height were derived from LiDAR. The date of each sampling event is indicated as well as the dates of phenological growth stages (GS) 31 and 45 (as attained by 50% of entries). For 90% of the lines, GS65 ranged from 22-Sep to 13-Oct (median GS65 date was 28-Sep). For AGB sampled at GS65, entries were sampled on the actual date they reached anthesis (or within two days of): therefore the lines were sampled on different dates and date is denoted "various". Correspondingly, the values of 3DVI, 3DPI, NDVI and height were interpolated between individual sampling events (i.e. 15-Sep, 25-Sep, 21-Oct and 25-Oct) for the GS65 date of each entry. CGR between stem elongation and anthesis was calculated as the difference in AGB divided by the duration of each period for each genotype (i.e. $t.ha^{-1}.day^{-1}$). CGR was calculated in the same way for the LiDAR biomass indices, 3DVI and 3DPI.

	Date<br>GS	8-Aug<br>31	16-Aug<br>–	22-Aug<br>–	6-Sep<br>45	15-Sep<br>–	25-Sep<br>–	various<br>65	21-Oct<br>–	25-Oct<br>–	CGR<br>31 to 65
AGB $(t.ha^{-1})$	M	1.20	–	–	–	–	–	8.69	–	–	0.14
	SD	0.29	–	–	–	–	–	1.94	–	–	0.03
	CV	0.24	–	–	–	–	–	0.22	–	–	0.22
3DVI $(-)$	M	5.10	6.06	7.12	8.86	9.08	10.46	10.55	11.18	11.25	0.10
	SD	1.02	1.35	1.25	1.26	1.18	1.27	1.15	1.47	1.40	0.02
	CV	0.20	0.22	0.18	0.14	0.13	0.12	0.11	0.13	0.12	0.21
3DPI $(-)$	M	0.39	0.58	0.85	1.29	1.68	1.83	1.88	1.96	1.87	0.03
	SD	0.18	0.28	0.37	0.46	0.50	0.47	0.46	0.49	0.49	0.01
	CV	0.47	0.48	0.43	0.36	0.30	0.26	0.25	0.25	0.26	0.24
NDVI $(-)$	M	0.62	0.61	0.66	0.73	0.78	0.76	0.75	0.64	0.60	–
	SD	0.08	0.09	0.10	0.09	0.06	0.06	0.07	0.10	0.11	–
	CV	0.12	0.15	0.14	0.12	0.08	0.08	0.09	0.16	0.19	–
Height (m)	M	0.34	0.43	0.49	0.63	0.74	0.81	0.83	0.87	0.88	–
	SD	0.07	0.10	0.09	0.09	0.10	0.10	0.11	0.10	0.11	–
	CV	0.20	0.23	0.18	0.15	0.13	0.13	0.13	0.12	0.12	–

Table 4. Summary of data from Yan17 experiment. Mean (M), standard deviation (SD) and coefficient of variation (CV) for above-ground biomass (AGB), green area index (GAI), leaf area index (LAI), 3D vegetation index (3DVI), 3D profile index (3DPI), normalized difference vegetation index (NDVI), crop height and crop growth rate (CGR). The 3DVI, 3DPI and crop height were derived from the LiDAR. CGR between stem elongation and anthesis was calculated as the difference in AGB divided by the duration (i.e. $t.ha^{-1}.day^{-1}$). CGR was calculated in the same way for the LiDAR biomass indices, 3DVI and 3DPI. The phenological growth stage (GS) and date is indicated for each sampling event.

	Date<br>GS	28-Aug<br>35	11-Sep<br>45	25-Sep<br>55	9-Oct<br>65	CGR<br>31 to 65
AGB $(t.ha^{-1})$	M	2.23	2.72	5.39	7.39	0.12
	SD	0.52	0.79	0.97	1.29	0.03
	CV	0.23	0.29	0.18	0.17	0.21
GAI $(-)$	M	1.33	1.97	2.39	2.87	–
	SD	0.49	0.55	0.55	0.66	–
	CV	0.37	0.28	0.23	0.23	–
LAI $(-)$	M	1.14	1.70	1.66	1.77	–
	SD	0.43	0.48	0.41	0.47	–
	CV	0.38	0.28	0.25	0.27	–
3DVI $(-)$	M	5.62	6.59	8.31	9.52	0.09
	SD	1.17	1.27	1.49	1.91	0.04
	CV	0.21	0.19	0.18	0.20	0.42
3DPI $(-)$	M	0.53	0.75	1.14	1.35	0.02
	SD	0.27	0.33	0.29	0.32	0.01
	CV	0.52	0.44	0.26	0.23	0.34
NDVI $(-)$	M	0.66	0.59	0.62	0.64	–
	SD	0.07	0.09	0.06	0.07	–
	CV	0.11	0.15	0.10	0.11	–
Height (m)	M	0.32	0.37	0.48	0.57	–
	SD	0.07	0.08	0.09	0.12	–
	CV	0.22	0.22	0.19	0.21	–
